# Evaluating prehospital trauma care in Stockholm from a gender perspective

**DOI:** 10.1186/1757-7241-22-S1-O2

**Published:** 2014-07-07

**Authors:** Rebecka M Rubenson Wahlin, Hanna K Lövbrand, Maaret K Castrén

**Affiliations:** 1 Department of Clinical Science and Education, Södersjukhuset, Karolinska Institutet, Stockholm, Sweden

## Background

Trauma is a common cause of death and permanent disability. With efficient and competent prehospital care many trauma-related deaths might be prevented. It is unknown whether gender influences the prehospital trauma care provided. However, research in other fields of emergency medicine, e.g. cardiovascular and intensive care has shown that women receive inferior care.

## Aim

The aim was to investigate whether there are gender differences in the prehospital care given to severely injured adult trauma patients in the Stockholm area.

## Method

Observational retrospective study based on a trauma registry and ambulance records. The study group consisted of 305 trauma patients, of which 230 were men (75.4%), and 75 were women (24.6%). Included were all adult (>15 years of age) trauma patients with an Injury Severity Score (ISS)>15 transported to Karolinska University Hospital in Stockholm between January 1^st^ and December 31^st^, 2008.

## Results

The background variables age, injury mechanism, ISS, and 30-day mortality were comparable between genders. There were no statistically significant gender differences in airway management, administration of oxygen, fluids, analgesics, and stabilization of neck and spine. Neither did on-scene time nor total prehospital time significantly differ between genders.

## Conclusion

This study indicates that there are no significant gender differences in the prehospital care given to severely injured adult trauma patients in the Stockholm area during 2008.
